# Novel Terpineol-Based Silver Nanoparticle Ink with High Stability for Inkjet Printing

**DOI:** 10.3390/nano15130955

**Published:** 2025-06-20

**Authors:** Aleksandrs Novikovs, Tamara Tsebriienko, Annamarija Trausa, Anete Berzina, George Chikvaidze, Dmitry Bocharov, Mohammad Yusuf Mulla, Juris Purans, Boris Polyakov

**Affiliations:** 1 Institute of Solid State Physics, University of Latvia, Kengaraga 8, LV-1063 Riga, Latvia; aleksandrs.novikovs@cfi.lu.lv (A.N.);; 2 Transport and Telecommunication Institute, Lauvas Str. 2, LV-1003 Riga, Latvia; 3 Smart Hardware, RISE Research Institutes of Sweden, Bio- and Organic Electronics, Södra Grytsgatan 4, Plan2, 602-33 Norrköping, Sweden; yusuf.mulla@ri.se

**Keywords:** silver nanoparticles, inkjet printing, terpineol, electrical conductivity

## Abstract

This study presents a novel silver nanoparticle ink formulation designed for inkjet printing applications using terpineol as an eco-friendly solvent and butylamine as a stabilizer to ensure stability, high conductivity, and compatibility with inkjet technology. Silver nanoparticles were synthesized using a modified one-pot method in the presence of highly effective stabilizers and surface modifiers such as oleic acid and oleylamine, resulting in uniform particles of less than 10 nm in size, which were then dispersed in a mixture of terpineol and butylamine. The resulting ink demonstrated exceptional stability over 85 days, maintaining optimal rheological properties for inkjet printing. The ink exhibited a perfect jetting performance. We were able to obtain silver conductive patterns reaching 81% of bulk silver conductivity. These results highlight the ink’s promise for scalable, sustainable manufacturing, combining environmental advantages with high-performance functionality.

## 1. Introduction

Metallic nanoparticles, such as silver, gold, and copper, are widely used in the fabrication of conductive films due to their excellent electrical conductivity. Among them, silver nanoparticles are preferable because of their relative cost-effectiveness, low sintering temperature, and oxidation resistance [1]. In addition, they exhibit distinctive optical, plasmonic, and antibacterial properties [2,3], making them highly suitable for a wide range of applications, including Radio Frequency Identification (RFID) antennas [4], Thin-Film Transistors (TFTs) [5], photosensors [6], Light-Emitting Diodes (LEDs) [7,8], and various types of sensors.

Ag-based conductive films can be prepared using various methods, depending on the target application and required resolution. Common approaches for preparing conductive patterns include screen printing [9], blade coating [10], dip coating [11], spin coating [12], and inkjet printing [13,14,15]. Among these, inkjet printing offers the high efficiency of material use, precise application over large areas, and simplicity in the production process at a low cost.

The formulation of the ink plays a crucial role in printing technology, as it directly determines the final properties and performance of the printed films. Key parameters such as the drying speed of the ink, the uniformity of the printed layer, and the electrical properties of the finished material are strongly influenced by the choice of chemical components used in the ink. Therefore, the success of the printing technology depends heavily on the careful design and optimization of the ink formulation [16]. Developing high-quality inks includes the identification of suitable solvents, surfactants, and binders [17,18]. To prevent nanoparticle agglomeration and precipitation and ensure stable silver nanodispersions, various substances are widely used, including surfactants like polyethylene glycol (PEG), neutral polymers such as polyvinylpyrrolidone (PVP) [19], ligands like citrate ions and thiol-based compounds [20,21], as well as ionic stabilizers [22]. Additionally, the choice of stabilizer influences whether the particles are hydrophilic or hydrophobic. This, in turn, affects how well the particles disperse in solvents of varying polarity and their ability to wet surfaces.

Conductive inks are generally classified into two main types based on the solvent used: organic solvent-based and water-based formulations. Organic solvent-based inks are widely favored for their ease of application, low viscosity, and rapid drying, which typically offer better processability than their water-based counterparts. However, the main disadvantages of solvent-based inks are their high cost and the toxicity of organic solvents, which limit their scalability for large-scale production [23]. In this context, terpineol emerges as a promising alternative solvent due to its availability, low cost, and environmental friendliness, as well as its suitable viscosity and low boiling point [24]. Despite these advantages, the use of terpineol has so far been mainly limited to the creation of graphene inks for inkjet printing. For instance, in the works [25,26], a surfactant-free graphene ink was prepared in a mixture of terpineol and cyclohexanone and optimized to obtain rheologies suitable for inkjet printing. The inks exhibited excellent stability and required only light sonication to avoid agglomeration. The optimum annealing conditions were 350 °C for 1 h, resulting in uniform films with significantly lower resistance compared to those based on surfactant-containing formulations.

Only a limited number of studies [27,28,29] have focused on the development of inkjet-printable formulations based on terpineol and metallic nanoparticles, particularly nanosilver. In Ref. [27], researchers reported a conductive ink composed of gold nanoparticles dispersed in alpha-terpineol [27]. By optimizing key printing parameters, the most important of which is the choice of solvent, it was possible to produce conductive lines with low resistivity (as low as 0.03 ohms/square) and an excellent surface morphology. A. Sels et al. [28] described highly conductive nanoinks containing 3–5 nm platinum nanoparticles dispersed in a toluene/terpineol mixture with a 15 wt% loading, optimized for inkjet printing. In addition, their lower sintering temperature of 200 °C allows for the use of flexible substrates for printing. In Ref. [29], inkjet inks containing colloidal silver and gold nanoparticles (up to 10 nm in diameter) dispersed in α-terpineol at a concentration of 10 wt% were developed. After sintering at 300 °C, a resistivity of 3 µΩ∙cm was achieved.

However, no data are currently available on the stability of terpineol-based Ag inks. The ink stability is a crucial factor for inkjet applications. A reliable ink formula prevents the aggregation and precipitation of silver nanoparticles during the printing process, maintaining consistent viscosity and surface tension.

This research aimed to develop a novel, environmentally friendly silver nanoparticle ink with high stability, specifically optimized for inkjet printing applications. Using terpineol as an environmentally friendly solvent and implementing complex stabilization using oleylamine, oleic acid, and butylamine, the research aimed to achieve stable dispersions, improve the rheological properties of the ink, and enable the fabrication of highly conductive printed patterns on glass substrates. This work addresses the key challenges related to ink stability and conductivity and contributes to the advancement of eco-friendly conductive ink technologies.

## 2. Materials and Methods

### 2.1. Materials

All the reagents were used as received without additional processing or purification. Silver nitrate (extrapure AgNO_3_ crystals, Sigma Aldrich, St. Louis, MO, USA) was used as the Ag precursor. Oleylamine (OA, C_18_H_37_N, Sigma Aldrich, technical grade 70%) was employed as the surfactant and reducing agent, while oleic acid (OAc, C_18_H_34_O_2_, Fisher Scientific, Waltham, MA, USA, laboratory-reagent-grade) acted as a surfactant to stabilize the nanoparticles and control their size and morphology. Toluene (Fisher Scientific, analytical-reagent-grade) and methanol (ACS reagents for synthesis) were used as solvents. Butylamine (Merck, Darmstadt, Germany, for synthesis) was used as an ink stabilizer, and terpineol (Sigma Aldrich, mixture of isomers, anhydrous) served as a solvent in the formulation of silver inks. Acetone (EMSURE, for analysis), isopropanol (EMSURE, for analysis), a 10% aqueous detergent solution (Hellmanex III, Sigma Aldrich), and deionized water were used to prepare the substrate for printing. Microscope glass slides (Marienfeld, Lauda-Königshofen, Germany) with an approximate thickness of 1 mm were used as substrates.

### 2.2. Methods

#### 2.2.1. Preparation of Silver Nanoparticles and Conductive Inks

In this work, we employed a slightly modified one-pot synthesis based on the method reported in [30] to produce narrow-distribution silver nanoparticles rapidly. In a typical run, 3.4 g of AgNO_3_ was introduced into a 100 mL solution consisting of oleic acid and oleylamine in a 9:1 ratio. This mixture was contained in a 200 mL three-neck round-bottom flask, which was equipped with a condenser, temperature controller, and magnetic stirrer. The system was thoroughly purged with nitrogen and maintained under continuous stirring no more than 120 rpm using a magnetic stirrer throughout the synthesis process. The reaction mixture was heated in an oil bath to 90 °C and was maintained at this temperature for 2 h to ensure the complete thermal decomposition of the silver nitrate and the formation of silver nanoparticles. Subsequently, the temperature was increased to 180 °C at a rate of 4 °C/min and was held for 5 min to promote uniformity in the size and shape of the nanoparticles. The resulting dark-brown solution was then cooled to room temperature. The synthesis yielded silver nanoparticles with a quantitative yield of 80%. Then, the product was purified using a precipitation/redispersion process. The reaction mixture was first diluted with toluene and precipitated with methanol in a 1:1 ratio, followed by centrifugation at 6000 rpm for 5 min. The precipitate was then redispersed in a toluene/butylamine mixture (10:1) and reprecipitated with methanol, followed by another centrifugation step. Conductive ink was prepared by dispersing Ag nanoparticles, obtained after the precipitation/redispersion process, in a solvent mixture of terpineol and butylamine (83:17 by volume). The resulting silver ink was a brown liquid containing up to 13.0 wt% of solid metal content. Before printing, the ink was filtered through a 0.1 µm pore size filter to remove any agglomerates or contaminants that could destabilize the printing.

For the Fourier Transform Infrared/Attenuated Total Reflectance (FTIR–ATR) spectroscopy, two types of samples were used: the Bu1 sample, prepared through precipitation/redispersion in a methanol and butylamine/toluene mixture (1:10), and model samples (Bu2–Bu6), which underwent 1 to 5 consecutive precipitation/dispersion cycles in a 1:1 butylamine/methanol mixture. This approach was employed to investigate the dynamics of the ligand exchange on the nanoparticle surface.

#### 2.2.2. Characterization of Silver Nanoparticles and Inks

Fourier Transform Infrared/Attenuated Total Reflectance (FTIR-ATR) spectroscopy was employed to investigate the chemical composition of the silver nanoparticle surfaces after synthesis and monitor changes in the coating agents following the precipitation/redispersion process. Spectra were acquired using a Vertex 80v FTIR spectrometer (Bruker Optik GmbH, Ettlingen, Germany) equipped with an ATRMax II Variable Angle Horizontal ATR Accessory (PIKE Technologies, Fitchburg, WI, USA) featuring a ZnSe crystal. The spectra were measured in the range of 500–3500 cm^−1^ with a spectral resolution of 2 cm^−1^. The pre-measurement sample preparation included the film formation of a 100 µL sample on the crystal through solvent evaporation.

Dynamic light scattering (DLS) was used to determine the hydrodynamic diameter of the nanoparticles. Measurements were conducted on a Litesizer^TM^ 500 particle (Anton Paar GmbH, Graz, Austria) size analyzer equipped with a 40 mW single-frequency laser diode (λ = 658 nm). Before the measurement, the samples were diluted 1:500 times in toluene and thoroughly de-dusted by filtration through membrane filters with an average pore diameter of 8 µm.

To monitor the surface plasmon resonance (SPR) peak of the silver nanoparticles as an indicator of the ink stability over time, UV-Vis spectroscopy was carried out using a SPECORD^®^ PC 210 spectrophotometer (Analytik Jena AG, Thuringia, Germany) with a variable wavelength in the range of 300–1000 nm. Measurements were performed in a glass cuvette with a 1 cm optical path length.

The morphological analysis and verification of the synthesized silver nanoparticles’ size were performed using transmission electron microscopy (TEM, FEI, Tecnai GF20, Hillsboro, OR, USA).

The temperature profile of dried ink at 100 °C was investigated by differential scanning calorimetry (DSC) and thermogravimetric analysis (TGA) using a Labsys evo thermogravimetric analyzer (Setaram Instrumentation, KEP Technologies, Caluire-et-Cuire, France). The experiments were carried out under the following conditions: an aluminum crucible was heated from 25 to 500 °C at a heating rate of 10 °C/min under air with a flow rate of 27 mL/min.

The viscosity and density of the conductive ink were measured using a Lovis 2000 M/ME micro-viscometer (Anton Paar GmbH, Graz, Austria), which operates based on the Rolling Ball Principle and is suitable for small fluid quantities (V = 2 mL). Measurements were performed at 22 °C.

The surface tension was measured using a Drop Shape Analyzer DSA100 (KRÜSS GmbH, Hamburg, Germany) at 22 °C by generating a 1 mL pendant drop at the tip of a needle. The ADVANCE for Drop Shape Analyzers software (Version 1.12-01) calculates the surface tension by fitting the theoretical drop profile to the recorded shape. The needle’s outer diameter (0.51 mm) was used as a reference.

Contact angle measurements were also performed using the Drop Shape Analyzer at 22 °C by placing 1 mL droplets onto cleaned substrates. The contact angle was recorded 5 s after the droplet detached from the needle tip. The substrates were cleaned before the measurements using the same procedure as that used for printing.

#### 2.2.3. Printing of Silver Conductive Inks, Their Thermal Treatment, and Characteristics of Printed Samples

Before printing, the microscopic glass substrates (13 × 20 mm) were successively treated with ultrasound in acetone, detergent, deionized water, and isopropanol. Each cleaning stage lasted 15 minutes. Printing was performed using a Dimatix 2850 inkjet printer (FUJIFILM Dimatix, Inc., Santa Clara, CA, USA) with DMC11610 cartridges (10 pL ink drop volume) and SAMBA cartridges (2.4 pL ink drop volume). A built-in drop watcher was used to visualize and evaluate the drop formation.

The printed droplet arrays were examined using a Nikon Eclipse LV150N optical microscope (Nikon Instruments, Tokyo, Japan) to investigate the printed drop network and the diameter, shape, and distribution of the printed drops in layers.

The annealing of the printed samples was conducted at temperatures ranging from 220 to 400 °C for 30 min in a laboratory oven with automatic temperature control and maintenance.

The surface morphology of the sintered nanoparticles was characterized using a scanning electron microscope (SEM, Thermo Scientific Helios 5 UX, Tokyo, Japan). The crystallographic structure of the conductive films was analyzed by X-ray diffraction (XRD) using a Rigaku MiniFlex 600 diffractometer (Rigaku, Tokyo, Japan) with Cu Kα radiation (λ = 1.5406 Å) over a Bragg angle 2θ range of 20° to 80° with a 0.02° step.

A scanning electron microscope (SEM, Thermo Scientific Helios 5 UX, Japan) was used to analyze the surface morphology of the sintered nanoparticles. The crystallographic structure of the conductive samples was analyzed using a Rigaku MiniFlex 600 diffractometer (Rigaku, Tokyo, Japan) equipped with an X-ray tube emitting Cu Kα radiation (λ = 1.5406 Å). Diffraction patterns were recorded over a 2θ Bragg angle range of 20° to 90° with a step size of 0.02°.

The film thickness was measured using a Dektak 150 Surface Profiler (Veeco, New York, NY, USA) equipped with a stylus of a 2.5 µm radius. The conductivity of the annealed films was evaluated by using a four-point probe system (Ecopia HMS-5000, Ecopia Corporation, Anyang-si, Republic of Korea).

## 3. Discussion

One of the key requirements for conductive inks is their stability during both storage and printing. Silver nanoparticles have high surface energy due to their small size and large surface area. This leads to the tendency of nanoparticles to agglomerate and flocculate to reduce their overall energy. Therefore, already at the stage of nanoparticle synthesis, it is important not only to achieve the desired particle size and shape but also to create conditions that provide long-term colloidal stability.

Oleic acid and oleylamine have been successfully employed as solvents, surfactants, and reducing agents. Both OAc and OAm are strong Lewis bases capable of forming coordination complexes with metal ions, which act as Lewis acids [31]. In addition, OAc and OAm can form acid–base complexes. These complexes also contribute to the synthesis process by functioning as both reducing and capping agents.

The resulting surface coating agent not only protects silver nanoparticles from oxidation and agglomeration but also serves as a dispersant, enabling their easy dispersion in a variety of polar solvents. The choice of solvent for ink formulation is therefore important, and nanoparticles that can be dispersed in a wide range of solvents simplify this procedure.

Methanol is commonly used in purification procedures to isolate and precipitate metal components from synthesized mixtures. However, it may also remove stabilizing agents from the surfaces of nanoparticles, adversely affecting their colloidal stability. In our study, during the purification of nanoparticles using pure methanol and toluene, we encountered gelation even after the first stage of precipitation/redispersion (Figure S1a).

To address this issue, we dispersed the same amount of silver in toluene with different amounts of additional stabilizers (Figure S1b–d). It was found that a solution of nanoparticles in toluene (1 mL) becomes homogeneous in the presence of 20 μL of butylamine or 40 μL of oleylamine (Figure S1b,d).

In the case of metal nanoparticles, ligand surface coatings are typically composed of insulating materials. As a result, residual ligands can negatively affect the electrical conductivity of the printed structures. Therefore, introducing butylamine as a stabilizer at the nanoparticle synthesis stage is advantageous. Owing to its lower boiling point (78 °C) compared to that of oleylamine (364 °C), butylamine can be more readily removed before and during the sintering process. Moreover, butylamine is more polar than oleylamine due to its shorter carbon chain and the presence of a primary amine group. In contrast, oleylamine contains a long hydrocarbon chain with a double bond, making it less polar. The presence of polar ligands, such as terpineol, on the surfaces of nanoparticles enhances their stability in polar environments.

**Figure 1:**
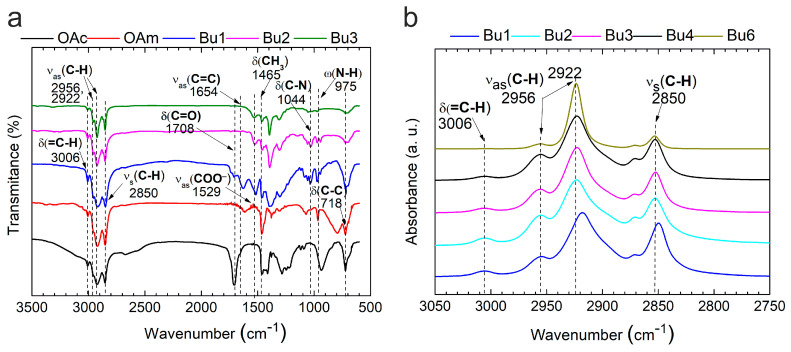
FTIR-ATR spectra (**a**,**b**) of oleylamine, oleic acid, and silver nanoparticles (Bu1) after one precipitation/redispersion cycle using a methanol and butylamine/toluene mixture (1:10), followed by one to five additional precipitation/redispersion cycles (Bu2–Bu6) with a methanol/butylamine mixture (1:1).

To gain a deeper understanding of the interactions between surfactants and nanocrystals during the precipitation/redispersion process, FTIR-ATR measurements were performed (Figure 1). This study included both nanoparticles prepared by the precipitation/redispersion procedure for the conductive ink formulation (sample Bu1) and a series of model samples (Bu2–Bu6). The preparation of these samples is described in Section 2.2.1.

Figure 1a shows the characteristic C−H vibrations observed for all the samples (Bu1–Bu6), which are also consistent with those found in OAc and OAm [32]. The symmetric C−H stretching vibrations appear at 2850 and 2956 cm^−1^, while the asymmetric stretching is observed at 2922 cm^−1^. In addition, bending vibrations are detected at 3006 cm^−1^ for the =C−H group and at 1465 cm^−1^ for the −CH_3_ group. These signals originate from the alkyl ligand fraction, indicating the presence of organic capping ligands on the surfaces of the nanoparticles. The C=O stretching vibration at 1712 cm^−1^ is characteristic of carboxylate (-COO) and was observed for both OAc and sample Bu1. Additionally, the 1529 cm^−1^ band is often associated with the asymmetric stretching vibration of the carboxylate group, particularly when they are coordinated to metal surfaces [33]. The fingerprint region, spanning approximately 1500 to 500 cm^−1^, exhibits a complex pattern of absorptions arising from numerous stretching and bending vibrations within the molecules. Due to this complexity, identifying specific functional groups is more challenging here than in the higher-wavenumber region. Nevertheless, a distinct peak at 718 cm^−1^ can be confidently assigned to the bending vibration of C−C bonds, indicating the presence of various organic compounds on the surfaces of the Ag nanoparticles [34].

To further investigate the extent of the ligand exchange on the nanoparticle surface during the purification process, we analyzed the stretching and bending vibrations of C–H bonds (δ(=C−H) at 3006 cm^−1^; ν_as_ C−H at 2956 and 2922 cm^−1^; ν_s_ C−H at 2850 cm^−1^), as well as the bending vibration of C–C bonds at 718 cm^−1^. Although OAm/OAc and butylamine ligands contain similar functional groups, they differ in the number of such bonds. As shown in Figure 1a,b, after modification with shorter ligands like butylamine, the abovementioned peaks decrease in intensity or disappear entirely, which likely indicates successful ligand exchange.

**Figure 2:**
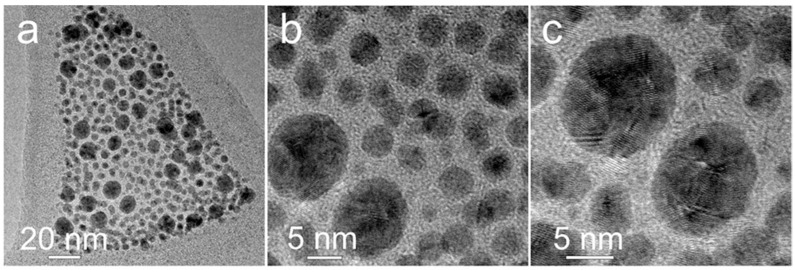
TEM images of Ag NPs obtained at different magnifications. The scale of each image (**a**–**c**) is indicated in the lower left corner of the corresponding figure.

Silver nanoparticles with a small size (<10 nm) exhibit a lower melting point and an enhanced atomic diffusion rate. At such small particle sizes, the ligand degradation temperature becomes a critical factor determining the formation of conducting pathways between metallic particles [35]. In addition, the nanoparticle size must be smaller than the nozzle diameter to prevent clogging during inkjet printing. According to [36], a sufficient condition for stable printing is that the particle size should be at least 50 times smaller than the nozzle diameter, which helps to avoid issues such as particle accumulation at the nozzle edge, droplet trajectory deviations, or nozzle blockage due to agglomeration. Thus, to assess the suitability of the synthesized silver nanoparticles for inkjet ink formulation, transmission electron microscopy (TEM) was used to evaluate their size. Figure 2a–c presents TEM images at different magnifications. The results confirm that the particles are spherical and do not exceed 10 nm in diameter, making them appropriate for printing processes and the fabrication of conductive patterns.

The stability of metallic nanoparticles is critical for conductive inks, as their flocculation can lead to nozzle clogging and interrupt the printing process. Therefore, selecting the appropriate solvent is essential to maintain the ink stability during both printing and storage. The Dimatix Materials Printer requires a solvent that evaporates slowly to ensure consistent droplet formation and smooth inkjet printing.

Solvent properties, such as viscosity and surface tension, are critical for controlling the movement of droplets from the nozzles to the substrate. These properties also affect the formation of convection and Marangoni flows, contributing to the coffee ring effect [37]. Binary solvent systems are often chosen to ensure uniform nanoparticle deposition, reduce defects, improve the print quality, and optimize the viscosity, surface tension, and drying time for precise inkjet printing.

For this study, terpineol was used as the primary solvent to disperse the nanoparticles and adjust the ink viscosity to meet the printing operational requirements. Butylamine was added as a stabilizing agent to prevent nanoparticle aggregation and ensure uniform dispersion. This solvent combination enhances the ink stability and improves the print quality.

**Figure 3:**
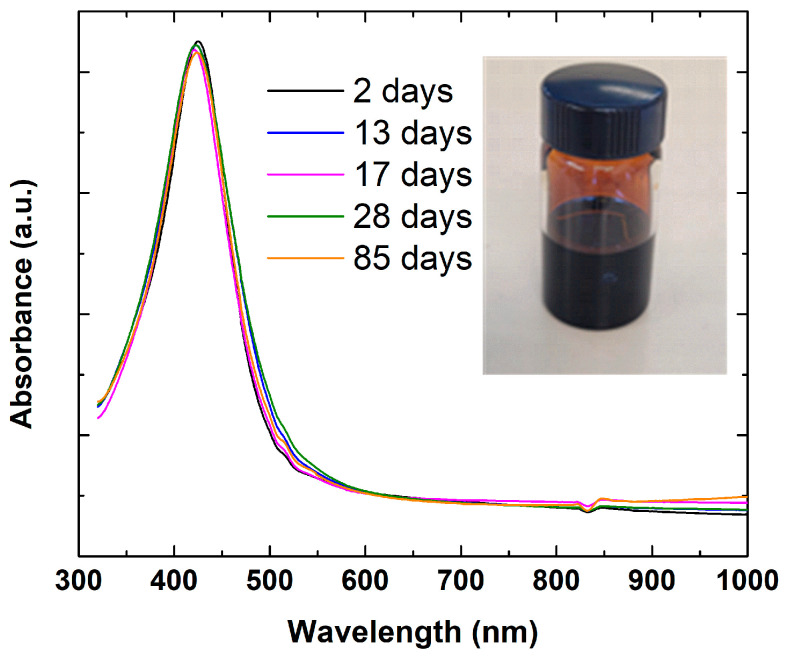
Normalized UV–Vis absorption spectra of silver inks recorded over different storage periods.

To assess the long-term stability of the prepared Ag inks, UV-Vis spectroscopy measurements were performed over 85 days (Figure 3). It is known that metallic nanoparticles possess free electrons that resonate with light waves, resulting in SPR. Small spherical silver nanoparticles give an SPR band extended in the range 350–500 nm with a peak position around 410–425 nm [38]. In this study, the presence of resonance absorption peaks at wavelengths around 421–425 nm was observed for all the samples, which was due to the excitation of SPR in silver nanoparticles (Figure 3). These results confirm the nanocrystalline nature of the particles and their low degree of polydispersity, with the particle size remaining fairly stable from the time of preparation up to 85 days of storage at room temperature.

**Figure 4:**
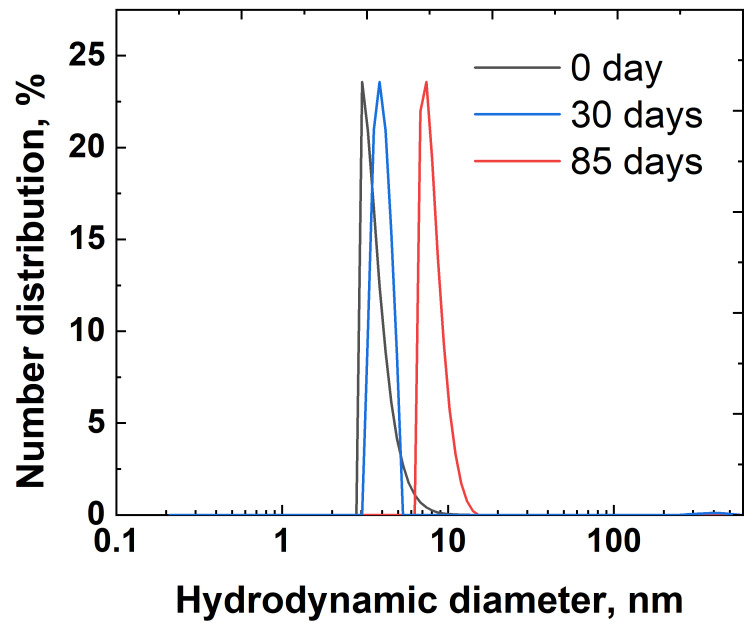
Hydrodynamic diameter of silver in inks, according to the DLS data over different storage periods.

Dynamic light scattering (DLS) analysis was employed to monitor the hydrodynamic size and stability of the Ag inks over 85 days of storage. The particle size distribution exhibited a single peak with slight polydispersity, indicating a relatively uniform population of nanoparticles (Figure 4). After 85 days of storage at room temperature, the average hydrodynamic diameter of the nanoparticles increased only slightly from ~5 nm to ~10 nm, which indicates the silver ink’s sufficient long-term stability.

**Figure 5:**
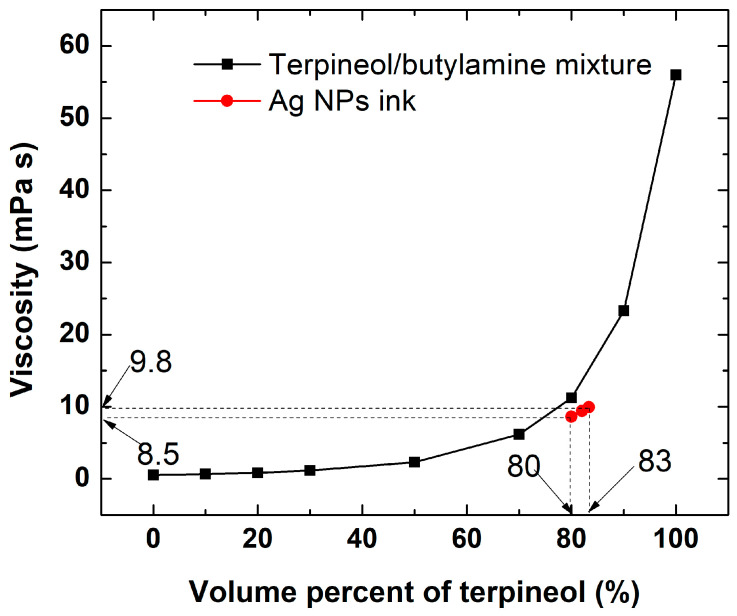
Viscosity calibration curve as a function of the volume percentage of terpineol in the terpineol/butylamine solvent system.

The rheological properties of conductive inks must meet the technological requirements of inkjet printing. Key factors like the surface tension and viscosity influence the velocity, size, and stability of the ejected droplets, as well as their spreading behavior on the substrate. The shape of these droplets directly influences the resolution and thickness of the printed patterns, which, in turn, determine their electrical performance. The Fujifilm Dimatix 2850 inkjet printer (DMC11610 cartridges) requires inks with a viscosity of 10–12 mPa·s, a surface tension of 27–33 mN/m, and a density around 1 g/mL [39]. SAMBA cartridges require slightly lower viscosity (4–8 mPa·s); to decrease the ink viscosity, the nozzle temperature can be slightly increased. To optimize the viscosity and surface tension of inkjet-compatible inks, we plotted a calibration curve of the viscosity versus the volume percentage of terpineol in the terpineol/butylamine mixture (Figure 5). The results show that the viscosity increases linearly with terpineol contents up to 60% and then rises sharply at higher concentrations.

Based on the calibration plot, an 80:20 terpineol/butylamine ratio was identified as optimal for achieving the desired viscosity. However, considering the presence of metal nanoparticles in the ink formulation, the solvent ratio was adjusted to 83:17 (terpineol/butylamine) to maintain the target viscosity (Figure 5). Table 1 shows the main physical parameters of the silver inks used in this work.

**Figure 6:**
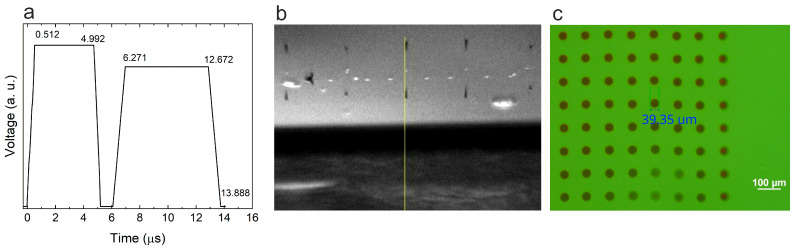
Inkjet printing process and droplet characterization: (**a**) jetting waveform; (**b**) fired drops from the printhead nozzles with spherical shape and minimal satellites (the yellow line indicating the vertical path of the falling droplets); (**c**) optical microscope image of pre-dried droplets (<40 μm) on glass substrate.

Before initiating the printing process, key parameters, including both the ejection voltage and waveform, droplet spacing, nozzle and plate temperature, and print height, were adjusted to ensure stable operation (Table 2, Figure 6a). To prevent temporary nozzle clogging and avoid the contamination of the substrate, cleaning cycles were performed after every 50 printed strips.

Printing was carried out in a layer-by-layer manner, forming 5 × 5 mm square patterns by depositing six successive layers of ink onto a glass substrate. The printed samples were then left to dry in ambient conditions overnight.

Figure 6b shows photos of droplets firing during printing. The droplets exhibit a spherical shape, follow a straight trajectory, and show minimal formation of satellite droplets. The printed samples have distinct edges, which also confirms the straightness of the droplets falling, the stable operation of the printing nozzles, and the satisfactory wetting of the glass substrate surface by liquid droplets.

A microscopic image of the pre-dried droplets on the substrate (Figure 6c) further demonstrates their spherical shape, with a diameter not exceeding 40 μm and no observable satellite droplets. These results confirm that the selected ink formulation is indeed suitable for providing inkjet print patterns.

**Figure 7:**
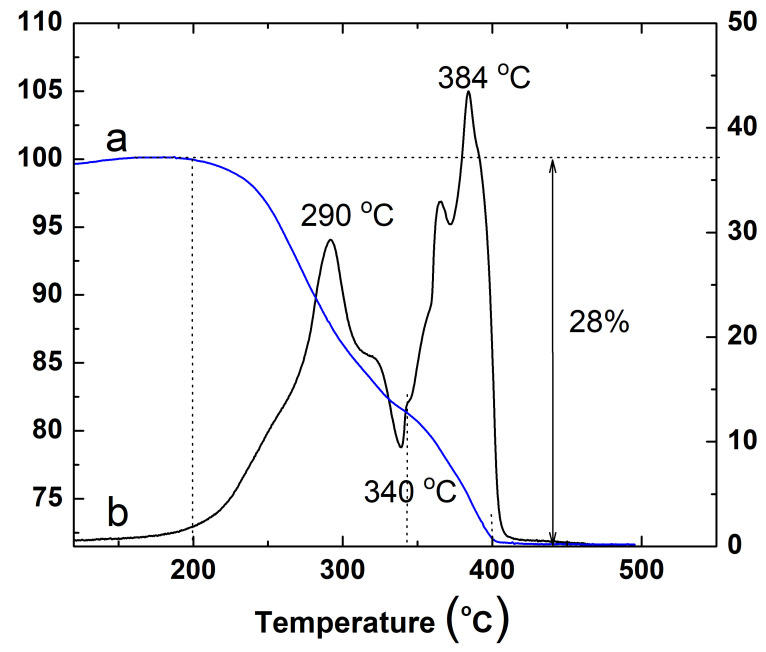
TGA (a curve) and DSC (b curve) diagrams of dried silver conductive ink.

The TG/DSC analysis of the conductive ink dried at 100 °C is presented in Figure 7. According to the TG data (the a curve in Figure 7), a continuous two-step mass loss of up to 28% is observed in the temperature range from 200 to 400 °C. The first stage, observed between approximately 200 and 350 °C, and the second, between 350 and 400 °C, are attributed to the gradual destruction of the organic ligand shell surrounding the silver nanoparticles. Above 400 °C, the residual mass remains stable, indicating the complete removal of volatile components.

The DSG results (the b curve in Figure 7) show two exothermic peaks: a large, sharp peak in amplitude at 384 °C and a small, broad peak at 290 °C. According to literature data [40], such exothermic signals in DSC during the heating of metal nanoparticles are typically associated with surface atom diffusion, the stabilization of the crystal structure, and grain boundary rearrangement as a result of their sintering. They may also indicate crystallization or recrystallization processes during the heating. Increased heat flow indicates significant surface diffusion. The result of the process is the formation of a continuous metal surface and agglomerates that improve the electrical properties of the material.

Additionally, the evaporation or desorption of organic species is usually an endothermic process. A broad endothermic peak around 340 °C in the DSC curve likely corresponds to the final desorption of surface ligands. This observation confirms that each sintering stage is preceded by the removal of the organic coating from the nanoparticle surface.

**Figure 8:**
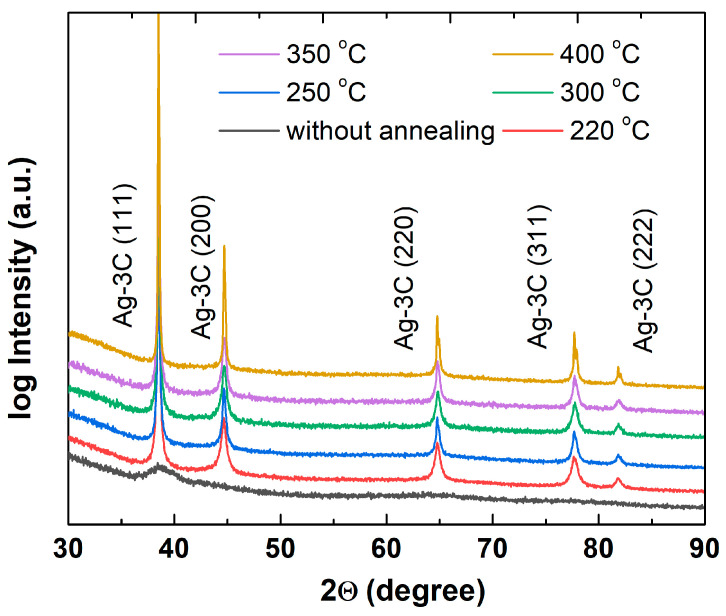
XRD patterns of silver nanoparticles annealed at various temperatures. Peaks of silver 3C face-centered cubic phase are marked. Intensity is shown in logarithmic scale.

The X-ray diffraction patterns of the annealed samples are presented in Figure 8. Distinct diffraction peaks are observed at 38.5°, 44.7°, 64.8°, 77.5°, and 81.2°, corresponding to the (111), (200), (220), (311), and (222) planes of face-centered cubic (fcc) silver. These reflection patterns confirm the single-phase composition of the samples and match the standard pattern for metallic silver (3C phase in Strukturbericht notation; JCPDS Card No. 04-004-5109 and Ref. [41]).

The crystallite sizes of the raw and annealed silver samples were estimated using Rietveld refinement analysis of the XRD spectra performed in Profex 5.3. software [42] (Table 3). A slight increase in the crystallite sizes of the samples was observed in the annealing temperature range of 220–350 °C. Notably, at 400 °C, the crystallite size increased nearly 3-fold compared to the unannealed sample. It is worth noting that the crystallite size for the as-prepared (raw) sample is consistent with the particle sizes determined by the DLS and TEM methods for ink (Table 1).

These observations are in good agreement with the DSC results, which revealed a significant increase in the heat flow between 350 and 400 °C, indicating an intensive crystallization process during the final stages of organic residue removal from their surfaces. Despite the observed grain growth, the crystalline phase remains unchanged: silver nanoparticles retain their stable fcc structure even at annealing temperatures as high as 400 °C.

**Figure 9:**
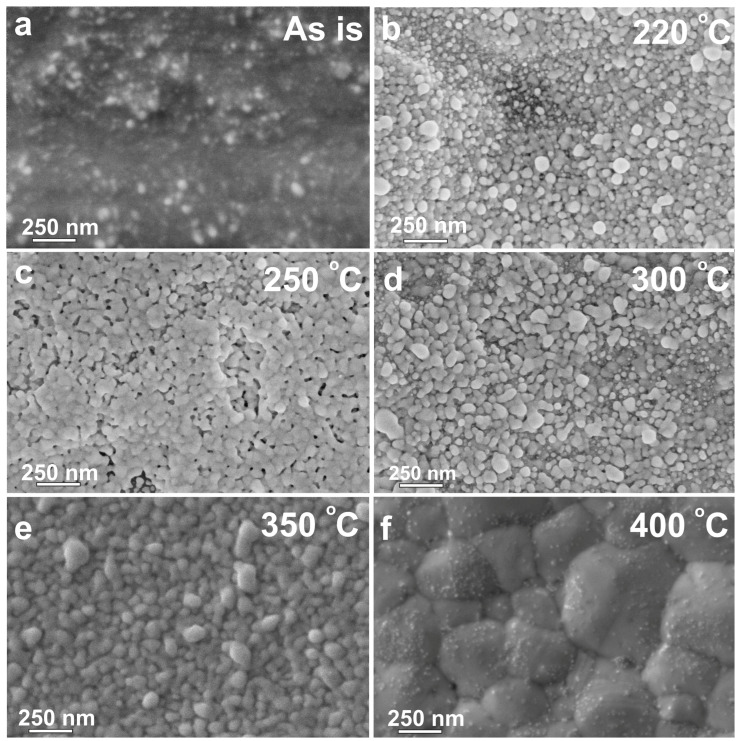
SEM images of printed silver nanoparticle patterns (**a**) without annealing and after 30 min of annealing at (**b**) 220 °C, (**c**) 250 °C, (**d**) 300 °C, (**e**) 350 °C, and (**f**) 400 °C.

**Figure 10:**
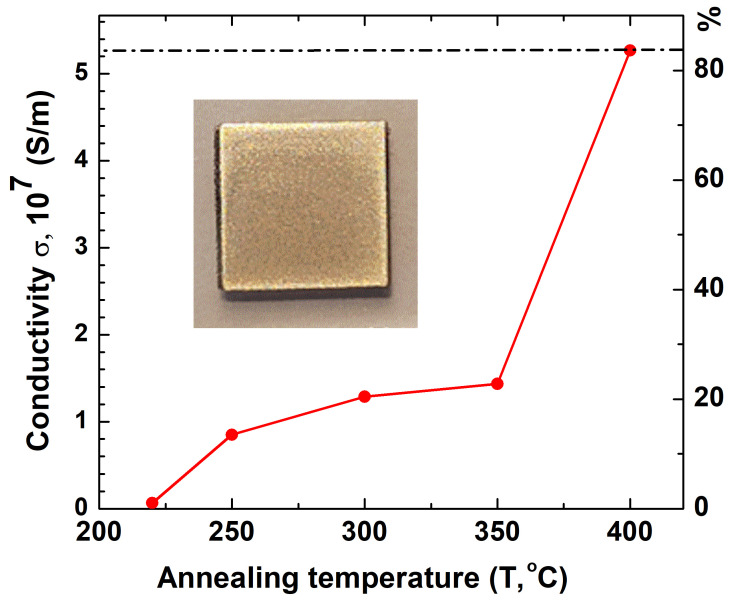
Conductivity of the printed patterns after sintering for 30 min at different temperatures. The inset shows a photograph of a sample annealed at 400 °C.

Upon the thermal sintering of the samples in the range of 220–400 °C for 30 min, the average film thickness decreased from 550 to 200 nm, primarily due to the removal of organic components from the ink. To investigate the effect of the sintering temperature on the film conductivity, the microstructures of the samples were investigated under different thermal treatment conditions. Figure 9 shows the surface morphologies of silver patterns annealed at 220, 250, 300, 350, and 400 °C. The corresponding conductivity values as a function of the sintering temperature are shown in Figure 10. All films exhibit a typical microstructure consisting of interconnected domains of sintered silver crystallites. The size of these domains varies significantly with the sintering temperature.

When heat treating was performed at temperatures above 220 °C, the silver particles underwent significant changes in both shape and size compared to their state before annealing (Table 3). In the samples annealed at 220–350 °C, domains of 50–100 nm in size were formed, while unheated particles had a size of up to 10 nm. Due to this, the samples treated at 220–350 °C already had a certain conductivity (Figure 10). Moreover, at 400 °C, a pronounced increase in the domain size up to 200–600 nm was observed, which correlates well with the significant growth of crystallites (Figure 9). The electrical conductivity of the samples annealed at 400 °C for 30 min increased to 5.21 × 10^7^ S/m, which is almost 83% of the electrical conductivity of bulk silver at room temperature equal to ≈ 6.30 × 10^7^ S/m (Figure 10) [43].

## 4. Conclusions

In this study, an environmentally friendly and stable silver ink suitable for inkjet printing was successfully developed. Using the modified one-pot synthesis method and a novel stabilization process using oleylamine, oleic acid, and butylamine, uniform silver nanoparticles with small sizes below 10 nm were obtained. The combination of terpineol as a solvent and butylamine as a stabilizer resulted in ink formulations that demonstrated excellent stability over an 85-day storage period and compatibility with inkjet printing requirements. The experimental results confirmed that the selected ink composition provides a reliable inkjet printing performance, defect-free printed patterns, and good electrical properties. Notably, annealing at 400 °C increases the film conductivity to 81% of bulk silver (5.1 × 10^7^ S/m), making this ink a promising candidate for the fabrication of highly conductive patterns. Additionally, the environmental benefits of terpineol highlight the potential for scaling this technology for sustainable large-scale production. Overall, the findings of this study contribute to the improvement in conductive inks, especially with terpineol-based formulations, paving the way for innovations in electronics and sensors.

## Figures and Tables

**Table 1:** Physical properties (average size, viscosity density, surface tension, and contact angle on washed glass substrates) of Ag inks.

Physical Parameters	Value
Average size of nanoparticles (nm) (according to TEM and DLS data)	≤10
Viscosity (mPa·s)	~10
Density (g/mL)	0.98–1.03
Surface tension (mN/m)	27
Contact angle on washed glass substrates (degrees)	18.0–21.5°

**Table 2:** Inkjet printing parameters.

Drop Spacing (µm)	Print Height (mm)	Cartridge Temperature (°C)	Substrate Temperature (°C)	Firing Voltage (V)	Jetting Frequency (kHz)
30	1.35	38	38	35	1.5

**Table 3:** Structural parameters of annealed silver nanoparticles estimated using Rietveld refinement analysis depending on annealing temperature.

Sample	Annealing Temp. (°C)	Lattice Parameter (nm)	Crystallite Size (nm)
1	-	0.4092 ± 0.0001	2 ± 1
2	220	0.4090 ± 0.0001	21 ± 1
3	250	0.4090 ± 0.0001	33 ± 1
4	300	0.4089 ± 0.0001	27 ± 1
5	350	0.4089 ± 0.0001	32 ± 1
6	400	0.4088 ± 0.0001	85 ± 1

## Data Availability

Data are contained within the article. Raw data are available upon request.

## Supplementary Materials

The following supporting information can be downloaded at https://www.mdpi.com/article/10.3390/nano15130955/s1. Figure S1: Photographs of silver nanoparticles solution after first precipitation/redispersion stage in different media.

## Abbreviations

The following abbreviations are used in this manuscript:

ATR	Attenuated Total Reflectance
DLS	Dynamic Light Scattering
DSA	Drop Shape Analyzer
DSC	Differential Scanning Calorimetry
FTIR	Fourier Transform Infrared
JCPDS	Joint Committee on Powder Diffraction Standards
LED	Light-Emitting Diode
NPs	Nanoparticles
OA	Oleylamine
OAc	Oleic Acid
PEG	Polyethylene Glycol
PVP	Polyvinylpyrrolidone
RFID	Radio Frequency Identification
SEM	Scanning Electron Microscopy
SPR	Surface Plasmon Resonance
TEM	Transmission Electron Microscopy
TGA	Thermogravimetric Analysis
TFT	Thin-Film Transistor
UV-Vis	Ultraviolet–Visible Spectroscopy
XRD	X-Ray Diffraction
wt%	Weight Percent
