# Supplementary material for: Novel Terpineol-Based Silver Nanoparticle Ink with High Stability for Inkjet Printing
Source: Nanomaterials (Basel). 2025 Jun 20;15(13):955. doi: 10.3390/nano15130955 (PMC12251367; doi:10.3390/nano15130955)
Supplement: Supplementary file 1 [file nanomaterials-15-00955-s001.zip › nanomaterials-3645230-supplementary.pdf]

Supplementary materials

a b c d

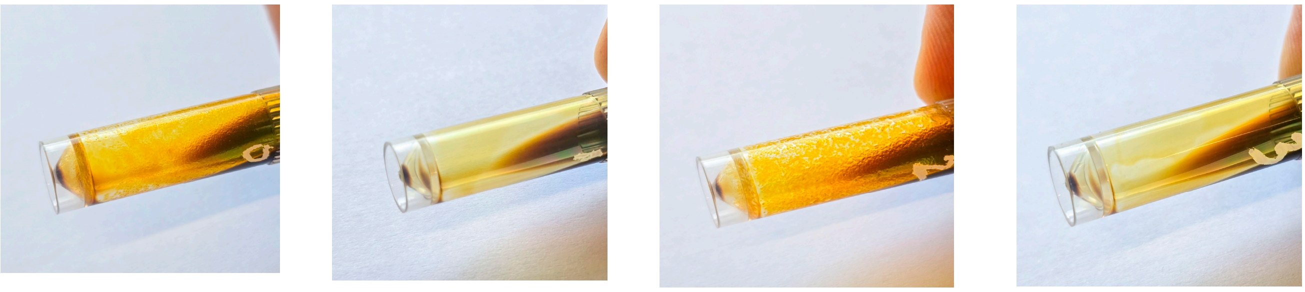

Figure S1. Photographs of silver nanoparticles solution after first precipitation/redispersion stage in different media: a) 1 mL toluene; b) 1 mL toluene with 20 μL of butylamine; c) 1 mL toluene with 20 μL of oleylamine; d) 1 mL toluene with 40 μL of oleylamine.
